# Chronic Non-Bacterial Osteomyelitis (CNO) in a Tertiary Center in Southern Italy: Response to Treatment and Outcome Stratification

**DOI:** 10.3390/children12040451

**Published:** 2025-03-31

**Authors:** Roberta Loconte, Rossella Donghia, Mariantonietta Francavilla, Giandomenico Stellacci, Carla Mastrorilli, Violetta Mastrorilli, Carlo Amati, Marcella Salvemini, Daniela Dibello, Giuseppe Ingravallo, Francesco De Leonardis, Stefano Palladino, Alberto Gaeta, Antonio Colella, Paola Giordano, Fabio Cardinale, Francesco La Torre

**Affiliations:** 1 Department of Pediatric, Giovanni XXIII Pediatric Hospital, 70126 Bari, Italy; roberta.loconte@asl.taranto.it (R.L.); carla.mastrorilli@policlinico.ba.it (C.M.); violetta.mastrorilli@libero.it (V.M.); fabio.cardinale@policlinico.ba.it (F.C.); 2 Data Science Unit, National Institute of Gastroenterology—IRCCS “Saverio de Bellis”, 70013 Castellana Grotte, Italy; rossella.donghia@irccsdebellis.it; 3 Department of Pediatric Radiology, Giovanni XXIII Pediatric Hospital, 70126 Bari, Italy; mariantonietta.francavilla@policlinico.ba.it (M.F.); giandomenico.stellacci@policlinico.ba.it (G.S.); stefano.palladino@policlinico.ba.it (S.P.); al.gaeta@libero.it (A.G.); 4 Department of Pediatric Orthopedic and Traumatology, Giovanni XXIII Pediatric Hospital, 70126 Bari, Italy; carlo.amati@policlinico.ba.it (C.A.); marcella.salvemini@alice.it (M.S.); daniela.dibello@policlinico.ba.it (D.D.); antonio.colella@policlinico.ba.it (A.C.); 5 Pathology Unit, Department of Precision and Regenerative Medicine and Ionian Area, University of Bari “Aldo Moro”, 70121 Bari, Italy; giuseppe.ingravallo@uniba.it; 6 Division of Paediatric Hematology-Oncology, University of Bari “Aldo Moro”, 70121 Bari, Italy; fdl111@libero.it; 7 Department of Interdisciplinary of Medicine, University of Bari “Aldo Moro”, 70029 Bari, Italy; paola.giordano@uniba.it

**Keywords:** autoinflammatory disorders, chronic non-bacterial osteomyelitis (CNO), chronic recurrent multifocal osteomyelitis (CRMO), bone pain, whole-body magnetic resonance imaging (WB-MRI), therapy, outcome, pain

## Abstract

**Background/Objectives:** Chronic non-bacterial osteomyelitis (CNO) is a rare autoinflammatory disease characterized by chronic sterile uni- or multifocal osteomyelitis. The treatment of CNO is mostly empirical and the outcome of the disease has not yet been standardized. The aims of this study were to correlate clinically active lesions with radiological signs of inflammation and to evaluate the outcomes in terms of symptoms and radiological signs with Whole Body Magnetic Resonance Imaging (WB-MRI) based on the treatment line used. **Methods:** A retrospective, observational cohort study of 20 CNO patients, recruited from a single tertiary center in southern Italy, was conducted. Patients included in the study were treated based on the “step-up” approach and were guided by the “treat-to-target” strategy as well as by the response to therapy. The outcome measure was stratified into four different groups, defined by a “Delphy consensus”, depending on the symptoms and the presence of bone lesions in WB-MRI, compared with the therapy carried out. **Results:** Pain was the most common presenting symptom of the disease. Only 15% of our patients reported long-term complications. WB-MRI was performed for each patient both at diagnosis and during follow-up. At onset, the site most affected by the disease was the tibia. All patients who reached a 5-year follow-up (30%, *n* = 6) achieved a complete disease remission. **Conclusions:** The standardized “step-up” treatment approach in our cohort proved effective in disease management with disease control or remission in nearly 90% of patients at one year from diagnosis.

## 1. Introduction

Chronic non-bacterial osteomyelitis (CNO), also known as chronic recurrent multifocal osteomyelitis (CRMO), is a rare autoinflammatory disease characterized by chronic sterile unifocal or multifocal osteomyelitis that clinically presents with recurrent episodes of pain and sometimes swelling of the bones and joints [1]. This disease mostly has a recurrent course with periodic exacerbations and remissions and its prognosis remains highly variable, but overall good [1,2].

### 1.1. Epidemiology

Epidemiological data suggest that the incidence of this disease is between 0.4 and 1/100,000; however, given the poor knowledge of the disease, this value is underestimated [3,4,5,6]. It seems to affect females more commonly, with an average age at onset between 9 and 11 years [6,7,8,9,10,11,12,13,14].

### 1.2. Clinical Characteristics

The main clinical feature is represented by bone pain, often associated with local swelling. However, the onset of the disease is usually insidious, characterized by modest and poorly localized bone pain, without local and/or systemic signs of inflammation. The symptoms can last months or years with a recurrent course. Only in rare cases does the disease start in an acute and significant manner characterized by intense pain, generalized malaise, weight loss and fever [7,8].

Sometimes the bone picture may be associated with skin manifestations (e.g., palmoplantar pustulosis, hidradenitis, acne and psoriasis) or other autoimmune diseases such as vasculitis (e.g., Takayasu arteritis), arthritis or inflammatory bowel disease(IBD) (e.g., Crohn’s disease) [7,8,10,11,15,16,17,18].

Bone lesions in CNO are usually multifocal and located at the level of the epiphyses and metaphysis of the long bones (particularly the tibia and femur) [7,9]. However, the entire skeleton can be involved with the disease and approximately one third of patients present vertebral involvement [7,9,11]. In the unifocal form, the main sites of localization are represented by the clavicle and the mandible [6,10,19,20].

### 1.3. Diagnosis

The diagnosis can be extremely complex given the non-specificity of the presentation, leading to an average delay of approximately 12 months from onset; the differential diagnoses include infections, onco-hematological diseases (leukemia, lymphoma and primary bone tumor), metabolic bone disorders and other inflammatory conditions such as Juvenile Idiopathic Arthritis (JIA) [8,10,21,22,23,24].

Physical examination is generally normal with pain in the site affected by the disease, but some patients present visible swelling or exhibit limping and/or difficulty walking, especially in cases of involvement of the pelvic bones and/or vertebrae [25,26].

#### 1.3.1. Laboratory Tests

Laboratory tests including inflammation markers (ESR and CRP) are generally normal or slightly increased [7,10,11]. Autoimmune investigations with dosages of AntiNuclear Antibody (ANA) and Human Leukocyte Antigen-B27 (HLA-B27) haplotype are often negative [8,10]. Further laboratory tests are used for differential diagnosis between neoplastic (LDH, uricemia), infectious (blood cultures, serologies) and metabolic bone diseases (vitamin D 25 (OH), ParaThyroid Hormone (PTH), and Alkaline Phosphatase (ALP), calcium, phosphorus).

The characteristics of the imaging of bone lesions, their distribution and complications are among the most important tools for the diagnosis and follow-up of CNO patients [6].

#### 1.3.2. Imaging

**Figure 1:**
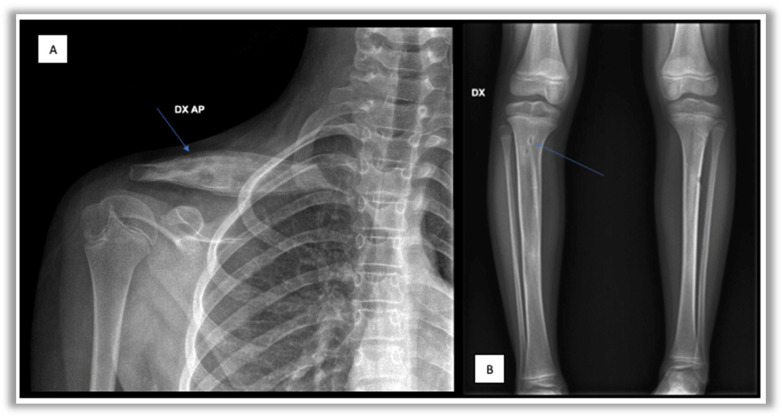
Radiographic images. (**A**) Right clavicular lesion;(**B**) right tibial osteolytic lesion.

**Figure 2:**
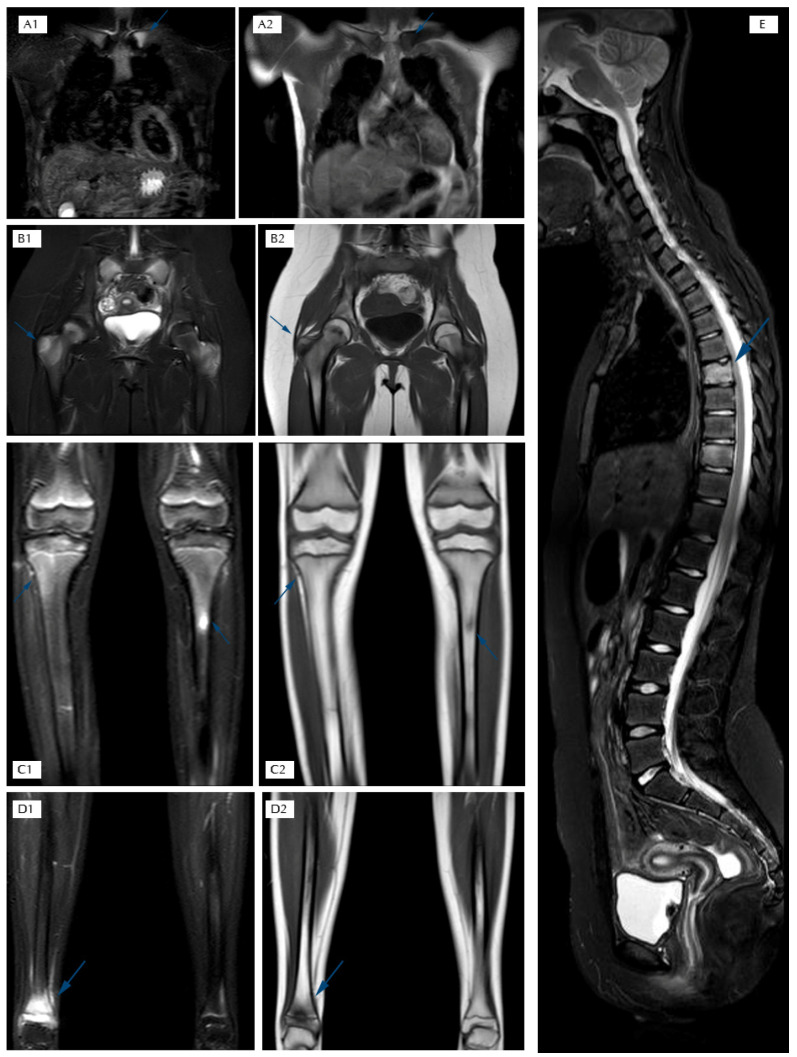
T2 STIR and T1 TSE sequences in the coronal plane: (**A1**–**D2**) STIR sequence in the sagittal plane of the spine; (**E**) STIR hyperintensity and T1 hypointensity are suggestive of bone marrow edema; (**A1**,**A2**) edema of the left clavicle in T2 STIR and T1 hypointensity; (**B1**,**B2**) edema of the greater trochanters in T2 STIR and T1 hypointensity; (**C1**,**C2**) edema of the proximal metaphysis and diaphysis of tibias in T2 STIR and T1 hypointensity; (**D1**,**D2**) edema of the distal metaphysis and epiphysis of the right tibia in T2 STIR and T1 hypointensity; (**E**) edema of two dorsal vertebral bodies in T2 STIR.

- Conventional radiography often represents the first-line investigation in patients with pain, but in the initial stages of the disease it is often negative. The first radiological changes are evident at the metaphyseal level near the growth cartilages, then an area of osteolysis surrounded by a sclerotic border can be highlighted in the evolution of the disease (Figure 1) [27].
- Bone scintigraphy with Technetium-99 in the initial phase of the disease is able to identify any hypercaptating but still silent bone focus [28]. In childhood, however, this test has important limitations such as hypercaptation at the level of growth cartilages and exposure to ionizing radiation.
- Magnetic Resonance Imaging (MRI) represents the most important diagnostic test to study inflammatory bone pathology in the initial phases of the disease, because of its high sensitivity for bone marrow edema even before the appearance of osteolysis and/or osteosclerosis [29,30,31]. Furthermore, this method does not involve exposure to ionizing radiation, and it has replaced bone scintigraphy in the diagnostic framework phase [29,30,31,32]. In CNO management, WB-MRI is usually performed and, thanks to its capability of monitoring the response to treatment, this represents the gold standard imaging test for diagnosis and follow-up. Through WB-MRI, it is also possible to visualize “silent lesions”, study the synovium, characterize the disease as unifocal or multifocal, recognize axial skeletal involvement, and identify the best site for performing bone biopsy [33]. Inflammatory lesions of CNO typically appear as hyperintense on T2-weighted sequences such as STIR (Short Tau Inversion Recovery) or TIRM (Turbo Inversion Recovery Magnitude) and hypointense on T1-weighted sequences (Figure 2) [34]. Acute lesions may also demonstrate restriction of the diffusion (DWI and ADC map sequences).

#### 1.3.3. Bone Biopsy

**Figure 3:**
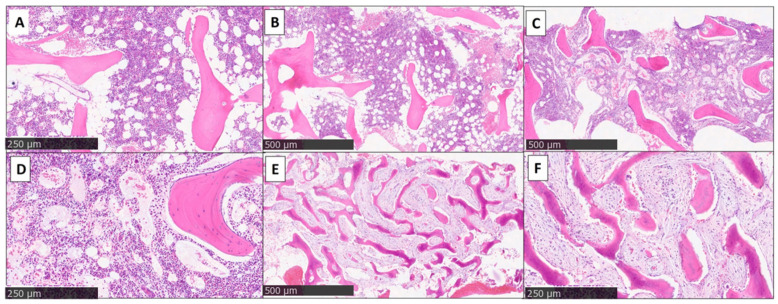
The histology of three cases (bone needle biopsies): (**A**,**B**) bone marrow with normal cellularity by age (“very early stage CNO”); (**C**,**D**) normal bone marrow associated with widespread sinusoidal dilatation (“early stage CNO”); (**E**,**F**) bone marrow with remarkable hypocellularity replaced by marked fibrosis, mild chronic inflammation and with accentuated remodeling of the bone trabeculae (“late stage CNO”).

Bone biopsy and its histological examination are usually mandatory to confirm the disease and to exclude neoplasms such as histiocytosis, lymphoma and other primary bone tumor or metastasis, especially in the case of disease with unifocal bone localization [35,36]. The results are not specific for CNO; infiltration of inflammatory cells, fibrosis, sclerosis and sometimes osteonecrosis are evident [6]. In the initial stage, the biopsy may be normal or show significant “early” lesions of an ongoing inflammatory process with a prevalence of neutrophil granulocytes, monocytes and macrophages (Figure 3). In the subsequent stages, the prevalence of lymphocytes and plasma cells can be observed, often with the permanence of monocytes and macrophages. The final stage is represented by bone sclerosis (Figure 3) [8,19]. However, the different stages can coexist with each other, showing both acute and chronic changes in the same tissue sample (Figure 3) [8,19].

### 1.4. Therapy

**Figure 4:**
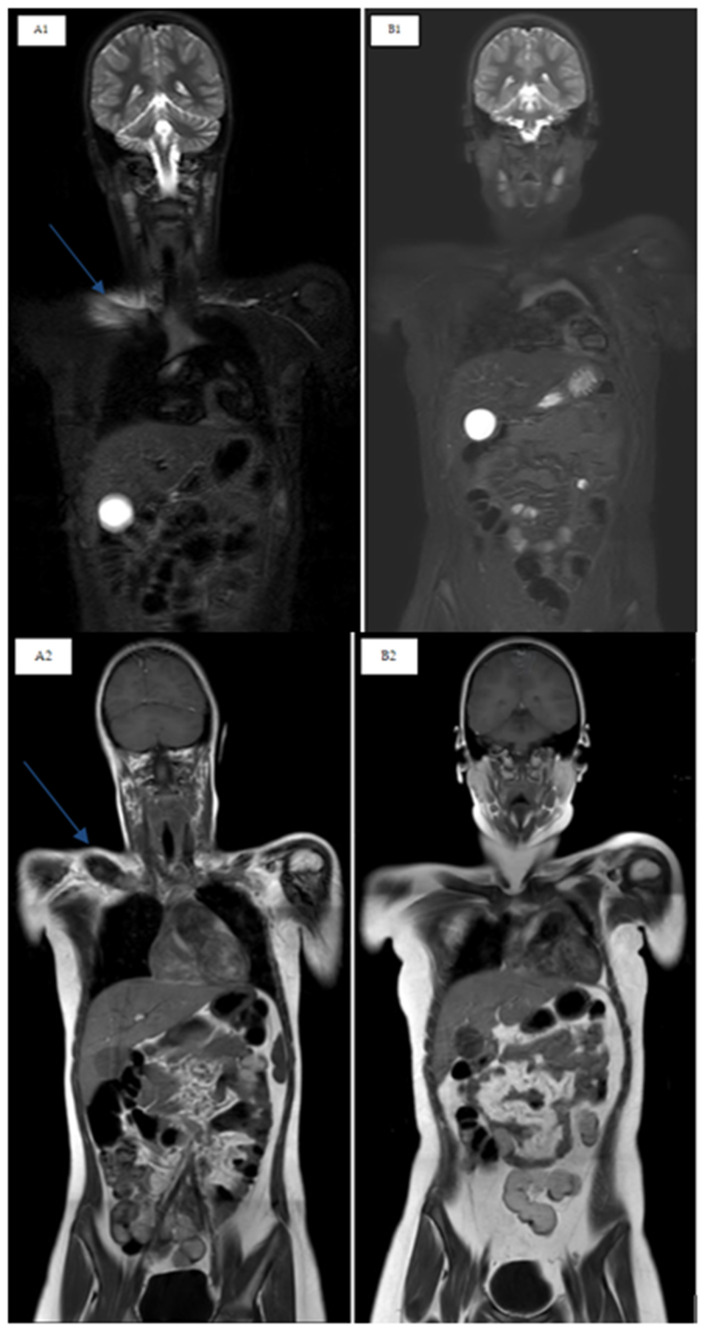
T2 STIR and T1 TSE sequences in the coronal plane. Right clavicle involvement in a CNO patient before and after pamidronate treatment. (**A1**,**A2**) Clavicle edema, at T0 pre-pamidronate in T2 STIR and T1 hypointensity; (**B1**,**B2**) regression of clavicle edema, at T12 post-pamidronate in T2 STIR and T1 hypointensity.

The aims of therapy are improving symptoms, preventing the progression of lesions and promoting bone health. The treatment of CNO, to date, has not yet been standardized. Nonsteroidal anti-inflammatory drugs (NSAIDs) represent the first line of treatment, proving their efficacy in a subgroup of patients [30,32]. However, the relapse rate remains high, and several studies show that 50% of patients treated with only NSAIDs relapse within 2 years [30,35]. Corticosteroids, bisphosphonates, methotrexate (MTX) and anti-TNFα (etanercept, adalimumab and infliximab) are used as second-line treatments with variable results [30,32]. Bisphosphonates and in particular pamidronate seem to have an important role in the management of this disease due to their rapid efficacy in bone remodeling by inhibiting osteoclastogenesis processes, reducing bone resorption and increasing bone mineralization [2,12,36,37,38]. Their efficacy in terms of the improvement of active lesions has also been reported [39]. In fact, in a time interval between 2 and 12 months it has been demonstrated that bone lesions were resolved in WB-MRI during follow-up among patients treated with pamidronate [33] (Figure 4).

### 1.5. Prognosis and Outcome

The prognosis is generally good. In most cases, CNO resolves without permanent damage, but if not promptly diagnosed and adequately treated it can be complicated by vertebral collapse, kyphosis, bone swelling and limb dysmetria [7,9]. The outcome of the disease has not yet been standardized. Historically, pain score has been used as a parameter for evaluating the response to therapy; however, it is strongly influenced by a subjective response [40,41]. Recently, a disease activity score (CNO Disease Activity Scale, CDAS) and a radiological score (ChRonic non-bacterial Osteomyelitis MRI Scoring, CROMRIS) have been proposed, in particular for clinical trials, although they are not universally used [42,43].

## 2. Objective

The aims of this study are to correlate clinically active lesions with radiological signs of inflammation and to evaluate the outcome in terms of symptoms and radiological signs at WB-MRI based on the treatment line used. We also evaluated the demographic, clinical therapeutic characteristics and the most significant findings of WB-MRI in pediatric patients affected by CNO recruited in a single tertiary center of southern Italy.

## 3. Methods

A retrospective, single-center, observational cohort study with CNO patients, diagnosed between January 2013 and October 2023 at the Department of Pediatrics of the “Giovanni XXIII” Pediatric Hospital of Bari, was conducted.

Format data analysis included gender, age at onset and at diagnosis, medical history (including family history), laboratory tests, imaging (X-ray, WB-MRI, bone scintigraphy), histology (bone biopsy), therapies with their side effects and long-term complications.

Inclusion criteria: patients under 18 years of age at onset, with unifocal or multifocal inflammatory bone lesions confirmed by WB-MRI (areas of hyperintensity in fat-saturated T2-weighted sequences) and with histopathological characteristics compatible with CNO (non-specific chronic inflammation, marrow fibrosis and osteonecrosis).

Exclusion Criteria: patients with SAPHO syndrome, infectious osteomyelitis, malignant bone disease (Ewing sarcoma, osteosarcoma, bone metastases or primary non-Hodgkin lymphoma of bone), hematological diseases (leukemia, lymphoma or Langerhans cell histiocytosis of bone), benign bone diseases (osteoid osteoma, osteoblastoma, chondroblastoma or cystic bone tumor) and patients over 18 years old of age at onset.

All patients were investigated with WB-MRI both at onset (T0) and after 6 (T6), 12 (T12), 24 (T24), 36 (T36), 48 (T48) and 60 (T60) months, depending on the duration of follow-up achieved by the individual patient.

The WB-MRI protocol consists of a whole-body STIR sequence and Turbo Spin Echo T1-weighted sequence in the coronal planes, acquired and merged with the Mobi View technique, followed by DWI and ADC map sequences in the axial planes in those patients who tolerated a prolonged examination. Contrast medium was administrated only in equivocal cases at diagnosis (e.g., unifocal aggressive lesion in differential diagnosis with a bone tumor).

Patients included in the study were treated based on the “step-up” and were guided by the “treat-to-target” strategy as well as by their response to therapy. Our protocol included the use of NSAIDs (ibuprofen or naproxen) as first-line treatments and bisphosphonate (pamidronate) as a second-line treatment started after one month of first-line therapy in the case of poor response or immediately at diagnosis with NSAIDs in case of vertebral involvement. Third-line treatments such as MTX were used in cases of the persistence of symptoms after three months from the start of pamidronate or in cases of disease relapse after this period. Anti-TNF biologics(etanercept) were finally used as a fourth-line treatment in case of symptom persistence after three months from starting MTX or in case of disease relapse during MTX therapy.

The outcome measure was stratified into four different groups depending on the symptoms (bone pain and/or swelling) and the presence of bone lesions in WB-MRI, compared with the therapy carried out (Table 1). The endpoints by “Delphy consensus” of four experts in the management of CNO were established.

### Statistical Analysis

The results were described by mean and standard deviation (M ± SD) for quantitative variables and by frequencies and percentages (%) for categorical variables. To test the association between independent groups evaluated a priori, the Chi-Square or Fisher test was used when necessary for categorical variables; on the contrary, the non-parametric Kruskal–Wallis test was used for continuous variables. For all analyses, a 95% confidence interval was evaluated and, to test the null hypothesis, two-tailed analysis was preferred with an error level of 0.05. The analyses were performed with STATA 17.0 Software (StataCorp. 2023. Stata Statistical Software: Release 18. College Station, TX, USA: StataCorp LLC.).

## 4. Results

### 4.1. Demographic Data and Clinical Characteristics

Twenty patients were enrolled and twelve (60%) of these were female. The mean age at onset was 9.12 years (range 7.13–11.11) while the mean age at diagnosis was 9.95 years (range 7.55–12.35) with a mean diagnostic delay of 10.7 months (range 0.4–20.73). Comorbidities and familiarity for autoimmune diseases in six (30%) and in two (10%) patients were recorded, respectively. No patients had a family history of CNO and/or other rheumatologic diseases. All of the patients had multifocal lesions but two of these (10%) presented unifocal lesions at onset before developing the others. The mean number of lesions was 6.75 (range 2.69–10.81) at onset and 2.35 (range −2.16–6.86) during follow-up (Table 2).Of the 20 patients enrolled, only two (10%) presented disease relapses after a variable time interval, eleven (55%) patients had inflammatory arthritis and two (10%) patients had skin involvement (one acne vulgaris and one plantar psoriasis) but these were different from the typical lesion of SAPHO syndrome.

The main clinical features at onset are included in Table 3. Pain was present in 19 (95%) cases and was nocturnal in 6 (30%); swelling was present in 15 (75%), functional limitation in 8 (40%) and lameness in 7 (35%) cases, respectively. Systemic symptoms such as general malaise and fever were reported in three (15%) and one (5%) patient, respectively. Only two patients had skin involvement (one acne vulgaris and one psoriasis) but these were different from those typical of Synovitis-Acne-Pustulosis-Hyperostosis-Osteitis (SAPHO) syndrome (Table 3). At onset, the clinical sites most involved were the tibia, tarsus, spine and clavicle, sometimes associated with swelling, functional impairment and limping. Long-term complications are reported in Table 4.

### 4.2. Laboratory Findings

Inflammation markers, such as CRP (mean value 5.63 mg/L, range −0.05–11.31) and ESR (mean value 26.55 mm/h, range 9.49–44.15), were increased, respectively, in four (20%) and six (40%) patients.

ANAs were positive only in one case, whereas extractable nuclear antigens (ENA), anti-native DNA antibodies (anti-dsDNA), HLA-B27 and rheumatoid factor (RF) were negative in the entire cohort. Other laboratory parameters such as complement proteins C3–C4 and immunoglobulins were also analyzed. Vitamin D 25(OH) deficiency was present in 13 (65%) patients. Bone biopsy and histological examination were performed in all patients (100%) and the results were compatible with the diagnosis of CNO.

### 4.3. Whole Body MRI

All patients were investigated with WB-MRI both at onset and at T6, T12, T24, T36, T48 and T60 months, depending on the duration of follow-up achieved by the individual patient.

At diagnosis (T0) the most involved sites were the tibia in thirteen (65%) patients (pts) and in six (30%) bilaterally; the femur in twelve (60%) pts and in five (25%) bilaterally; the tarso-metatarsalin in nine (45%) pts and in four (20%) bilaterally; the spine in eight (40%) pts; the acetabulum in five (25%) pts, the same as the fibula and clavicle which occurred bilaterally in two (10%) and one (5%) patients, respectively. The pelvis, humerus and radius were present in four (20%) pts, and bilaterally in one (5%) patient each. The scapulae and ulna were present in two (10%) patients and occurred bilaterally in one (5%) each. The carpo-metacarpal, sternum and ribs were present in one (5%) patient. At diagnosis, none of our patients presented mandible involvement and the joints were involved in 10 patients (50%) (Table 5).

Table 6 shows the total number of lesions detected with WB-MRI, at diagnosis and during disease course, for each patient enrolled in the study.

### 4.4. Treatment

Patients were treated according to the “step-up” protocol previously described. In particular, all patients (100%) were treated with NSAIDs (ibuprofen or naproxen), sixteen patients (80%) with pamidronate of which two (10%) were treated since diagnosis for vertebral involvement, nine (45%) patients with MTX, three (15%) pts with etanercept and one (5%) with prednisone for severe presentation, respectively. Furthermore, all our patients, especially during therapy with bisphosphonates, underwent supportive therapy with calcium carbonate and vitamin D.

### 4.5. Outcome

Table 7 shows the outcome based on the four different groups defined in Table 1 and calculated for each individual patient enrolled in the study.

The data showed that all patients who reached T60 (30%, *n* = 6) achieved disease remission off medication considered as the absence of clinical signs, absence of lesions highlighted by WB-MRI and the end of therapy.

In particular, our study is focused on the 12 months after diagnosis, because this was achieved by the entire cohort with the exception of a single patient enrolled in October 2023.

Evaluating the outcome after one year of treatment (T12) with our “step-up” protocol, 21.05% (*n* = 4) of patients achieved “remission off medication” (group 4), 26.32% (*n* = 5) “control off medication” (group 3), 42.11% (*n* = 8) “control on medication” (group 2) and only 10.5% (*n* = 2) “poor control” (group 1). The outcome trend, together with the statistically significant therapy (*p* < 0.001), seems to confirm the efficacy of our “step-up” therapeutic strategy (Table 7 and Table 8).In fact, the patients in our cohort are in disease control or remission in nearly 90% of cases at one year from diagnosis.

Furthermore, describing the lesions still observable in WB-MRI at T12, the spine showed a high prevalence in group 1 (100%, *n* = 2/2), but was lower in group 2 (37.5%, *n* = 3/8) and absent in groups 3–4, with an overall *p* = 0.03 (Table 8).

## 5. Discussion

We presented a retrospective, single-center study, describing the demographic, clinical, radiological, therapeutic and outcome characteristics of a cohort of 20 pediatric patients affected by CNO diagnosed in the period between January 2013 and October 2023.

The data concerning clinical and epidemiological characteristics are comparable to other studies already published [14,25,37].

Unlike other studies, in our case no patient presented an association with other inflammatory diseases such as IBD [8,11,44,45,46]. Six patients (30%) presented other comorbidities such as Rolandic epilepsy or Hashimoto’s thyroiditis. Two patients (10%) presented a family history of autoimmune disease, but none for CNO and/or other rheumatological diseases.

The prognosis of this disease is generally good; long-term complications are reported in few cases and include limb dysmetria, residual swelling and vertebral collapse [8,11]. In our cohort, only three patients (15%)reported complications (one patient reported vertebral collapse, one leg discrepancy and another one residual swelling), with a lower rate than other published studies [37,44].

Our study confirms that pain is the most common presenting symptom of the disease, associated with bone swelling and/or arthritis. Systemic symptoms are instead reported in a small percentage of patients [8,11,37]. In our cohort, general malaise and fever were reported in three (15%) and one (5%) patients, respectively.

All patients underwent WB-MRI at diagnosis and during follow-up, confirming that it is the gold standard tool for the diagnosis and monitoring of patients, for their response to treatment and for correlating clinically active lesions with radiological signs of inflammation.

All our patients had multifocal bone involvement but two of these (10%) presented unifocal lesions (one clavicle and one pelvis) at onset before developing the others.

Unlike many other studies in which the main site is represented by the clavicle, in our study the tibia was the site most involved at onset, and it was present in thirteen patients (65%) and in six (30%) who were bilateral [25,44]. The other sites involved were the same as the other studies [7,11,44,45,46].

Recently, two radiological scores have been proposed to improve the interpretation of images in correlation with the disease; however, both have limitations because they have not yet been validated and are not applicable in our cohort of patients with a follow-up of approximately 10 years [42,43].

All our patients made bone biopsies, and all results were compatible with the diagnosis of CNO (without the typical histological neoplastic abnormalities and with the infiltration of lymphocytes and plasma cells compatible with non-specific chronic inflammation and/or marrow fibrosis and/or osteonecrosis).

CNO therapy has not yet been standardized, even though all studies in the literature confirm the use of NSAIDs as first-line treatment; however, several studies show that 50% of patients treated only with NSAIDs relapse within 2 years [30,35].

Among the classic DMARDs, MTX and salazopyrine are often used for their anti-inflammatory effects. Nevertheless, their success is extremely variable. In particular, for MTX clinical remission from 20% to 66% has been reported [3,8,10,14].

Biological drugs such as TNF-α blockers have been used lately for cases of CNO which having failed in other treatments, and this is based on the evidence of increased serum TNF-α concentrations in patients with active disease as well as the role of TNF in bone damage and inflammation [3,30]. Studies over the years have evaluated the efficacy of TNF-α blockers in CNO (etanercept, adalimumab and infliximab), and have shown an efficacy ranging between 46 and 89%, with clinical remission in 3 months [3,10,36,47]. More recent studies have confirmed these positive observations, reporting an efficacy ranging between 50 and 90.9% of cases [8,19,37,48,49].

Corticosteroids are sometimes used for short periods in diseases with very severe presentation. Steroids are not recommended as a long-term treatment due to their well-known side effects [44].

**Figure 5:**
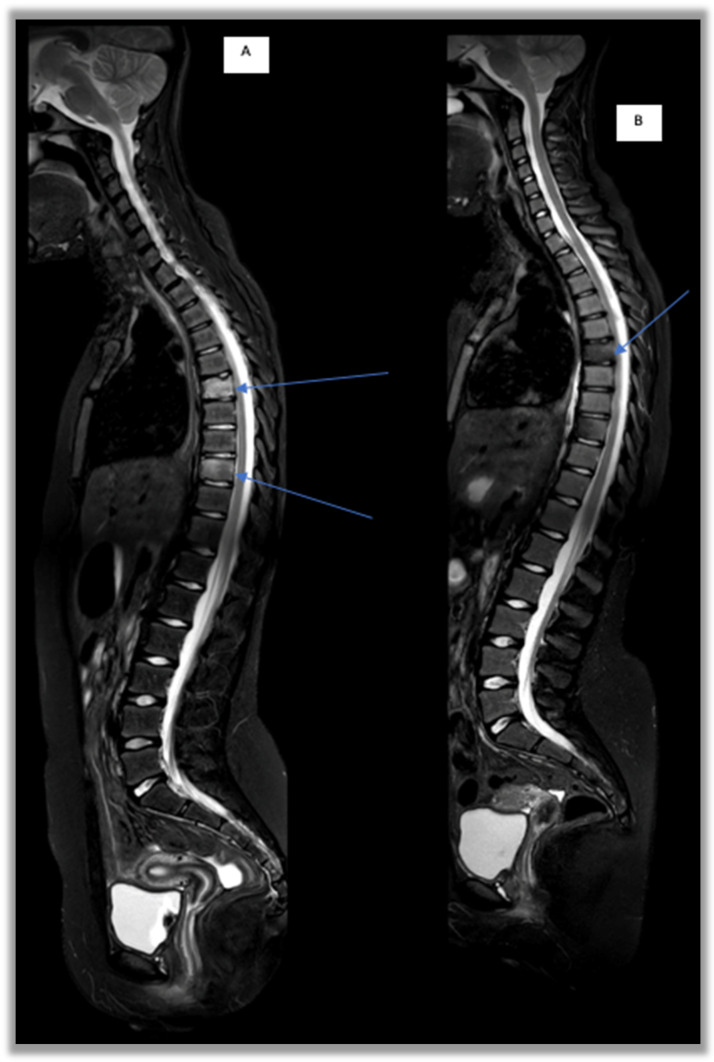
STIR sagittal sequences of the spine. D6 and D9: vertebral involvement in a CNO patient before and after pamidronate treatment. (**A**) D6 and D9 vertebral edema, MRI-WB T0 pre-pamidronate; (**B**) regression of bone marrow edema at T12 follow-up post-pamidronate.

In several recent studies, an increase in the use of bisphosphonates is reported; these play an important role in bone remodeling by inhibiting osteoclastogene processes, reducing bone resorption and increasing bone mineralization [19,50]. In particular, given its rapidity of action, pamidronate is the most frequently used. More recent retrospective studies confirm the previous results, with remission achieved in 69.4–91% of patients [8,37,48,49,50,51,52] and in particular in cases of vertebral involvement [40,53] (Figure 5). More recently, a remarkably high response rate in vertebral lesions after pamidronate treatment has been shown, with 82.3% of lesions resolving completely, suggesting that pamidronate could be proposed as a first-line treatment in cases of spinal and mandibular involvement [50,52].

In our center, patients were treated according to a gradual strategy based on “step-up” and guided by “treat-to-target” by the response to therapy. Our protocol included the use of NSAIDs (ibuprofen or naproxen) as first-line treatments and bisphosphonate (pamidronate) as a second-line treatment started after one month of first-line therapy in cases of poor response or immediately at diagnosis with NSAIDs in cases of spine involvement. Third-line drugs such as methotrexate were used in cases of the persistence of symptoms after three months from starting pamidronate or in cases of relapse of the disease after this period. Biological anti-TNF etanercepts were finally used as fourth-line treatments in cases of the persistence of symptoms after three months from the start of MTX or in cases of disease relapse during MTX therapy.

In particular, all patients (100%, *n* = 20) were treated with NSAIDs (ibuprofen or naproxen), sixteen patients (80%) with pamidronate of which two (10%) were treated since diagnosis for vertebral involvement, nine (45%) with MTX, three (15%) with etanercept and one (5%) with prednisone for severe presentation at disease onset were treated, respectively. Furthermore, all our patients underwent supportive therapy with calcium carbonate and vitamin D.

We also evaluated the outcomes by stratifying patients into four different groups depending on their symptoms and the lesions in WB-MRI in comparison to the class of drugs used. These endpoints were established by “Delphy consensus” of four experts in the management of CNO.

The analysis showed that all patients who reached T60 (30%, *n* = 6) achieved complete disease remission. In particular, our study was focused on the 12 months from the diagnosis of the disease, a timing reached by the entire cohort with the exception of a single patient enrolled in October 2023. At the outcome assessment after one year of treatment (T12) with our “step-up” protocol, 21.05% (*n* = 4) of patients achieved “remission off medication” (group 4), 26.32% (*n* = 5) “control off medication” (group 3), 42.11% (*n* = 8) “control on medication” (group 2) and only 10.5% (*n* = 2) “poor control” (group 1) (Table 8). This seems to confirm the efficacy of our “step-up” therapeutic strategy with disease control or remission in nearly 90% of patients at one year from diagnosis.

Finally, we described the bone lesions still observable in WB-MRI and statistically significant at T12 depending on the outcome group. Of these, the spine showed a high prevalence in group 1, a lower prevalence in group 2 and was absent in groups 3 and 4 (Table 8). This result confirms the worse outcome for vertebral involvement in CNO, considering the greater possibility of long-term complications (e.g., vertebral collapse), and indicating in these patients the need for third- (MTX) and fourth-line (anti-TNF) treatments immediately at the time of diagnosis to obtain disease control in a shorter time.

## 6. Conclusions

In our study clinical, laboratory, imaging, treatment and outcome characteristics of a cohort of 20 CNO patients have been described. Despite the limitations of the study, which are its retrospective analysis and the small size of our cohort, this number, although small, is significant for this rare disease. Furthermore, even if it is a retrospective observational study, the homogeneity of diagnostic and therapeutic management within the same center allowed us to correlate the treatment with the outcome.

Therapy in CNO has not yet been standardized, but our accurate “step-up” treatment approach, guided by the “treat-to-target” strategy and response to therapy, has been shown to be effective in disease management with disease control or remission in nearly 90% of patients at one year from diagnosis.

Analyzing the outcome, we evaluated stratifying patients into four different groups defined by a “Delphy consensus”, and it emerged that in our cohort all patients who reached T60 (30%, *n* = 6) achieved complete disease remission.

However, further studies are needed to understand the disease and to reach a universally accepted diagnostic and therapeutic protocol.

## Figures and Tables

**Table 1:** Outcome measures in CNO established through “Delphy consensus”.

	Group 1 Poor Control on Medication	Group 2 Control on Medication	Group 3 Control off Medication	Group 4 Remission off Medication
Symptoms	YES	NO	NO	NO
WB-MRI lesions	YES	YES	YES	NO
Therapy	YES	YES	NO	NO

**Table 2:** Epidemiological and clinical characteristics of our CNO patients.

Parameters *	M ± SD or %
Gender (%)	
Female	12 (60.00)
Male	8 (40.00)
Age at onset (Years)	9.12 ± 1.99
Age at diagnosis (Years)	9.95 ± 2.40
Time between onset and diagnosis (Months)	10.70 ± 10.03
Familiar autoimmune disease (Yes) (%)	2 (10.00)
Comorbidity (Yes) (%)	6 (30.00)
Reactivations (Yes) (%)	2 (10.00)
Involvement (Yes) (%)	
Skin	2 (10.00)
SAPHO	0 (0.00)
Joints	11 (55.00)
Multifocal	20 (100.00)
N. lesions at onset	6.75 ± 4.06
N. current lesions	2.35 ± 4.51

* As mean and standard deviation (M ± SD) for continuous variables and as frequency and percentage (%) for categorical variables.

**Table 3:** Clinical characteristics at onset of our cohort.

Parameters *	%
Pain	19 (95.00)
Night pain	6 (30.00)
Swelling	15 (75.00)
Thermotact	5 (25.00)
Fever	1 (5.00)
General malaise	3 (15.00)
Weight loss	0 (0.00)
Plantar psoriasis	1 (5.00)
Lameness	7 (35.00)
Functional limitation	8 (40.00)
Acne	1 (5.00)
Pustulosis	0 (0.00)
Hidradenitis suppurativa	0 (0.00)

* As frequency and percentage (%) for categorical variables.

**Table 4:** Long-term complications reported in our cohort.

Parameters *	%
Vertebral collapse	1 (5.00)
Leg discrepancy	1 (5.00)
Residual swelling	1 (5.00)
Functional limitation	0 (0.00)
Plantar psoriasis	0 (0.00)
Acne	0 (0.00)
Pustulosis	0 (0.00)
Hidradenitis suppurativa	0 (0.00)
Other	0 (0.00)
Total	3 (15.00)

* As frequency and percentage (%) for categorical variables.

**Table 5:** Bone involvement at diagnosis in our cohort.

Parameters *	%
Jaw (%)	0 (0.00)
Unilateral	0 (0.00)
Bilateral	0 (0.00)
Spine (Yes) (%)	8 (40.00)
Clavicle (%)	5 (25.00)
Unilateral	4 (20.00)
Bilateral	1 (5.00)
Sternum (Yes) (%)	1 (5.00)
Ribs (%)	1 (5.00)
Unilateral	1 (5.00)
Bilateral	0 (0.00)
Shoulder blades (%)	2 (10.00)
Unilateral	1 (5.00)
Bilateral	1 (5.00)
Humerus (%)	4 (20.00)
Unilateral	3 (15.00)
Bilateral	1 (5.00)
Ulna (%)	2 (10.00)
Unilateral	1 (5.00)
Bilateral	1 (5.00)
Radius (%)	4 (20.00)
Unilateral	3 (15.00)
Bilateral	1 (5.00)
Carpus—Metacarpus (%)	1 (5.00)
Unilateral	1 (5.00)
Bilateral	0 (0.00)
Femur (%)	12 (60.00)
Unilateral	7 (35.00)
Bilateral	5 (25.00)
Acetabulum (%)	5 (25.00)
Unilateral	5 (25.00)
Bilateral	0 (0.00)
Tibia (%)	13 (65.00)
Unilateral	7 (35.00)
Bilateral	6 (30.00)
Fibula (%)	5 (25.00)
Unilateral	3 (15.00)
Bilateral	2 (10.00)
Tarsus—Metatarsus (%)	9 (45.00)
Unilateral	5 (25.00)
Bilateral	4 (20.00)
Pelvis (%)	4 (20.00)
Unilateral	3 (15.00)
Bilateral	1 (5.00)
Joints (Yes) (%)	10 (50.00)

* As percentage (%) for categorical variables.

**Table 6:** Number of lesions in WB-MRI in our cohort.

Parameters	T0 (*n* = 20)	T6 (*n* = 19)	T12 (*n* = 19)	T24 (*n* = 12)	T36 (*n* = 9)	T48 (*n* = 8)	T60 (*n* = 6)
ID 1	4	4	2	2	4	1	0
ID 2	9	4	0				
ID 3	7	6	11	4			
ID 4	2						
ID 5	3	1	0				
ID 6	6	5	4	2	1	1	0
ID 7	1	3	1				
ID 8	10	8	6	5			
ID 9	7	5	4	3	1	0	0
ID 10	8	5	1	2	4		
ID 11	1	2	0				
ID 12	3	4	4	2			
ID 13	9	5	2	1	1	0	0
ID 14	9	1	5				
ID 15	16	14	13	11	5	5	0
ID 16	5	3	2	2	1	0	0
ID 17	4	2	0				
ID 18	13	6	9	10	6	2	
ID 19	4	4	2	2	1	1	
ID 20	14	20	20				

**Table 7:** Outcomes of CNO patients in our cohort.

Parameters	Group 1	Group 2	Group 3	Group 4
T0 (*n* = 20)	100% (*n* = 20)	0% (*n* = 0)	0% (*n* = 0)	0% (*n* = 0)
T6 (*n* = 19)	52.63% (*n* = 10)	36.84% (*n* = 7)	10.53% (*n* = 2)	0% (*n* = 0)
T12 (*n* = 19)	10.53% (*n* = 2)	42.11% (*n* = 8)	26.32% (*n* = 5)	21.05% (*n* = 4)
T24 (*n* = 12)	0% (*n* = 0)	66.67% (*n* = 8)	33.33% (*n* = 4)	0% (*n* = 0)
T36 (*n* = 9)	11.11% (*n* = 1)	55.56% (*n =* 5)	33.33% (*n* = 3)	0% (*n* = 0)
T48 (*n* = 8)	0% (*n* = 0)	37.5% (*n* = 3)	25% (*n* = 2)	37.5% (*n* = 3)
T60 (*n* = 6)	0% (*n* = 0)	0% (*n* = 0)	0% (*n* = 0)	100% (*n* = 6)

**Table 8:** Parameters stratified at T12 into different outcome groups.

Parameters *	Group 1 (*n* = 2)	Group 2 (*n* = 8)	Group 3 (*n* = 5)	Group 4 (*n* = 4)	*p*
Spine (Yes) (%)	2 (100.00)	3 (37.50)	0 (0.00)	0 (0.00)	0.03
Joints (Yes) (%)	1 (50.00)	4 (50.00)	0 (0.00)	0 (0.00)	0.11
Number of lesions	8.50 ± 3.53	7.12 ± 6.51	2.40 ± 1.52	0.00 ± 0.00	0.006 ^¥^
Therapy					<0.001
None	0 (0.00)	0 (0.00)	5 (100.00)	4 (100.00)	
Ibuprofen	0 (0.00)	0 (0.00)	0 (0.00)	0 (0.00)	
Pamidronate	0 (0.00)	0 (0.00)	0 (0.00)	0 (0.00)	
MTX	1 (50.00)	7 (87.5)	0 (0.00)	0 (0.00)	
Etanercept	0 (0.00)	0 (0.00)	0 (0.00)	0 (0.00)	
MTX+etanercept	1 (50.00)	1 (12.5)	0 (0.00)	0 (0.00)	
Supportive therapy					0.11
Nothing	0 (0.00)	0 (0.00)	1 (20.00)	1 (25.00)	
Ca carbonate + Vit.D	2 (100.00)	6 (75.00)	2 (40.00)	0 (0.00)	
Vitamin D	0 (0.00)	20 (25.00)	2 (40.00)	3 (75.00)	

* As mean and standard deviation (M ± SD) for continuous variables and as frequency and percentage (%) for categorical variables. Chi-Square or Fisher test when necessary; ^¥^ Kruskal–Wallis test.

## Data Availability

The data analyzed during the current study are available from the corresponding author on reasonable request. The data are not publicly available due to privacy and technical limitations.

## Abbreviations

CNO: Chronic Non-bacterial Osteomyelitis; CRMO: Chronic Recurrent Multifocal Osteomyelitis; WB-MRI: Whole Body Magnetic Resonance Imaging; IBD: Inflammatory Bowel Disease; JIA: Juvenile Idiopathic Arthritis; ESR: Erythrocyte Sedimentation Rate; CRP: C-Reactive Protein; ANA: AntiNuclear Antibody; HLA-B27: Human Leukocyte Antigen-B27; LDH: Lactate DeHydrogenase; PTH: ParaThyroid Hormone; ALP: Alkaline Phosphatase; STIR: Short Tau Inverse Recovery; TIRM: Turbo Inversion Recovery Magnitude; NSAIDs: Nonsteroidal Anti-Inflammatory Drugs; MTX: Methotexate; TNF: Ymor Necrosis Factor; CDAS: CNO Disease Activity Scale; CROMRIS: ChRonic non-bacterial Osteomyelitis MRI Scoring; SAPHO: Synovitis, Acne, Pustulosis, Hyperostosis and Osteitis syndrome; ENA: Extractable Nuclear Antigens; dsDNA Ab: anti-Double Stranded DNA antibodies; RF: Rheumatoid Factors; DMARD: Disease-Modifying Anti-Rheumatic Drug.
